# One-Step Purification of Recombinant Human Amelogenin and Use of Amelogenin as a Fusion Partner

**DOI:** 10.1371/journal.pone.0033269

**Published:** 2012-03-19

**Authors:** Johan Svensson Bonde, Leif Bulow

**Affiliations:** Department of Pure and Applied Biochemistry, Center for Chemistry and Chemical Engineering, Lund University, Lund, Sweden; University of Crete, Greece

## Abstract

Amelogenin is an extracellular protein first identified as a matrix component important for formation of dental enamel during tooth development. Lately, amelogenin has also been found to have positive effects on clinical important areas, such as treatment of periodontal defects, wound healing, and bone regeneration. Here we present a simple method for purification of recombinant human amelogenin expressed in *Escherichia coli*, based on the solubility properties of amelogenin. The method combines cell lysis with recovery/purification of the protein and generates a >95% pure amelogenin in one step using intact harvested cells as starting material. By using amelogenin as a fusion partner we could further demonstrate that the same method also be can explored to purify other target proteins/peptides in an effective manner. For instance, a fusion between the clinically used protein PTH (parathyroid hormone) and amelogenin was successfully expressed and purified, and the amelogenin part could be removed from PTH by using a site-specific protease.

## Introduction

Enamel is the hardest tissue in the vertebrate body and is made up from highly arranged hydroxyapatite crystals. During enamel formation the enamel matrix is secreted from special epithelial cells called ameloblasts and around 90% of the organic content of the developing enamel matrix is comprised of the extracellular protein amelogenin. The suggested function of amelogenin in enamel formation is to direct the growth of the hydroxyapatite crystals, which are highly elongated along the c-axis [1]–[3]. In the enamel matrix amelogenin is proteolytically processed by matrix metalloproteinase-20 and kallikrein-4, which together with alternative splicing of amelogenin transcripts creates a complex mixture of amelogenin poly peptides. As the enamel matures amelogenin is removed from the matrix by proteolytic degradation, and mature enamel is almost completely inorganic [4]. Much depending on the complexity of the enamel matrix the exact mechanism of amelogenin during enamel formation remains to be revealed.

Lately amelogenin has been detected also in non-dental tissue, such as brain and the hematopoietic system [5]. *In vitro* and *in vivo* experiments have shown that amelogenin, apart from acting as a structural protein in biomineralization, also appears to have signal molecule like properties [6]–[7]. Porcine enamel matrix derivate (EMD), containing extracted amelogenins, have been found to regenerate periodontal tissues like periodontal ligament (PDL), cementum and alveolar bone [8], and is used clinically in dentistry to treat periodontal defects [9], and also in the treatment of leg ulcers [10]. A positive effect on bone regeneration has also been seen in rat femur and skull [11]–[12], and amelogenin has been reported to have positive effects on angiogenesis and wound healing in animal models [10], [13]–[15]. So far the clinical applications of amelogenin are limited to treatment of periodontal defects and leg ulcers, but increased clinical use may be expected in the future [16]. To explain the apparent regenerative effect of amelogenin it has been proposed that amelogenin may have several different functions and that certain splicing variants have distinct effects on different cell types. Amelogenin seems to enhance the proliferation of PDL cells [17]–[18], regulate genes associated with cementoblasts [19]–[20], and have been found to induce chondrogenic and osteogenic phenotype in embryonic muscle fibroblasts [21]. Apart from use as a therapeutic, amelogenin has also been proposed to promote creation of new biomaterials, such as coatings on titanium implants, giving further applications to amelogenin proteins [22].

Amelogenin has the ability to form supramolecular aggregates (termed nanospheres) [23], and this property appears to be important for the biological activity during enamel formation [24]. Mutations in amelogenin, found in the disease *Amelogenesis imperfect*, causing deformed enamel, have been found to affect amelogenin aggregation properties such as nanosphere size *in vitro* [25]–[27]. This implies a close connection between nanosphere formation and biologic activity of amelogenin. Larger amelogenin assemblies, as a result of further aggregation of amelogenin nanospheres, have also been reported [28]–[29]. The aggregation behavior of amelogenin is related to its characteristic solubility properties, with the best solubility observed in the acidic area, low solubility at physiological pH, followed by a higher solubility in the basic area [30]–[31].

Recombinant amelogenin produced in *Escherichia coli* was first described by Simmer et al. [30], using the most common splicing variant of murine amelogenin. The described method for isolation of recombinant amelogenin, which has been used extensively in subsequent reports, comprises: cell disruption by sonication under denaturating conditions (6 M guanidine hydrochloride), centrifugation, repeated ammonium sulphate precipitations, centrifugation, followed by reversed phase (C4) chromatography. The purified protein is obtained in aqueous acetonitrile at a reported yield of 4–11 mg/ liter cell culture. Expression levels of recombinant amelogenin can be improved in *E. coli* by using an N-terminal histidine tag [32].

The aim of this study was to find a method of isolating untagged recombinant amelogenin without using acetonitrile or chaotropic agents, preferably feasible to do in a large scale. We managed to utilize the solubility properties of amelogenin to efficiently separate recombinant human amelogenin from host cell proteins in a one-step procedure using intact *E. coli* cells. A procedure to use the same method for purifying other recombinant proteins/peptides is demonstrated.

## Results

### Creating an amelogenin expression vector

**Figure 1:**
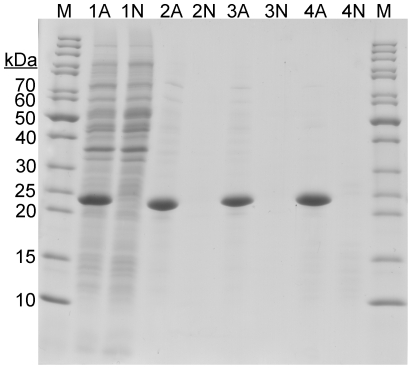
SDS-PAGE analysis of samples from different stages of purification of recombinant amelogenin from *E. coli* cells. Lanes marked with (A) represent samples from cells expressing amelogenin and lanes marked with (N) refers to negative control cells harboring empty expression vector. Lanes marked with (1) represent whole cell samples after cultivation. Lanes marked (2), (3) and (4) represent samples after different treatments of the intact cells. (2): soluble fractions of cells sonication in 3% HAc. (3): soluble fractions after heat treatment of the sonicated samples in lanes 2A and 2N. (4): soluble fractions of cells directly heat treated at 80°C. This generates the most pure amelogenin. Lanes marked (M) contain molecular weight markers.

For expression of recombinant amelogenin a synthetic gene encoding the most common splicing variant of human amelogenin (Swissprot Q99217, isoform 1, excluding the signal peptide), codon optimized for *E. coli*, was successfully created. Amelogenin was expressed from a pET11 plasmid vector (Novagen) in *E. coli* BL21 (DE3) cells after cultivation in shake flasks and high levels of amelogenin could be observed when total cell proteins were analyzed by SDS-PAGE (Figure 1).

### Amelogenin purification

After cultivation the amelogenin expressing cells were harvested by centrifugation. Cell disruption by sonication in solutions with different pH generated different amounts of soluble amelogenin in the cleared cell lysates. Sonication in buffers with physiological pH resulted in a low concentration of soluble amelogenin, but this could be improved by lowering the pH. The presence of contaminating host cell proteins in the cleared lysates was also affected by the pH in the sonication buffer, with higher amounts present at physiological pH. Sonication of the amelogenin expressing cells in 3% acetic acid yielded a soluble amelogenin, and at the same time also precipitated the major fraction of *E. coli* proteins (Figure 1, lane).

To test the stability of amelogenin in the cleared lysate obtained after sonication in 3% HAc the lysate was subjected to heat treatments at 60 or 80°C. Analysis of the soluble fractions after the heat treatment showed that amelogenin was still soluble after 20 minutes at 80°C. No reduction in soluble amelogenin concentration or other negative effects of the heat treatment could be observed.

Since sonication is not very feasible in large scale, disruption of intact amelogenin expressing cells using heat treatment was tested. Intact harvested cells suspended in 3% HAc were heat treated at 80°C for 20 minutes, without prior treatment. Cell debris and precipitated proteins were removed by centrifugation, and the soluble fraction was analyzed by SDS-PAGE. This treatment generated an amelogenin solution with very low amounts of host cell proteins. The purity of the amelogenin was higher when only heat treatment was used for cell lysis/purification, compared to when sonication was used, alone or in combination with heat treatment (Figure 1). Control experiments with cells harboring empty pET11 vector were performed and showed very low amount of soluble proteins after treatment at low pH and high temperature.

Variations in the acid/heat treatment purification procedure can be made and still obtaining comparable results. HAc concentrations between 0.5 and 5% were successfully tested, showing an increase in yield at the higher concentrations, but a slightly lower purity. The volume of HAc used was successfully tested between 60 and 600 ml for cells from one liter of cultivation (21 g wet weight cells), with the total yield increasing with increasing volume HAc (data not showed).

### Characterization of purified amelogenin

**Figure 2:**
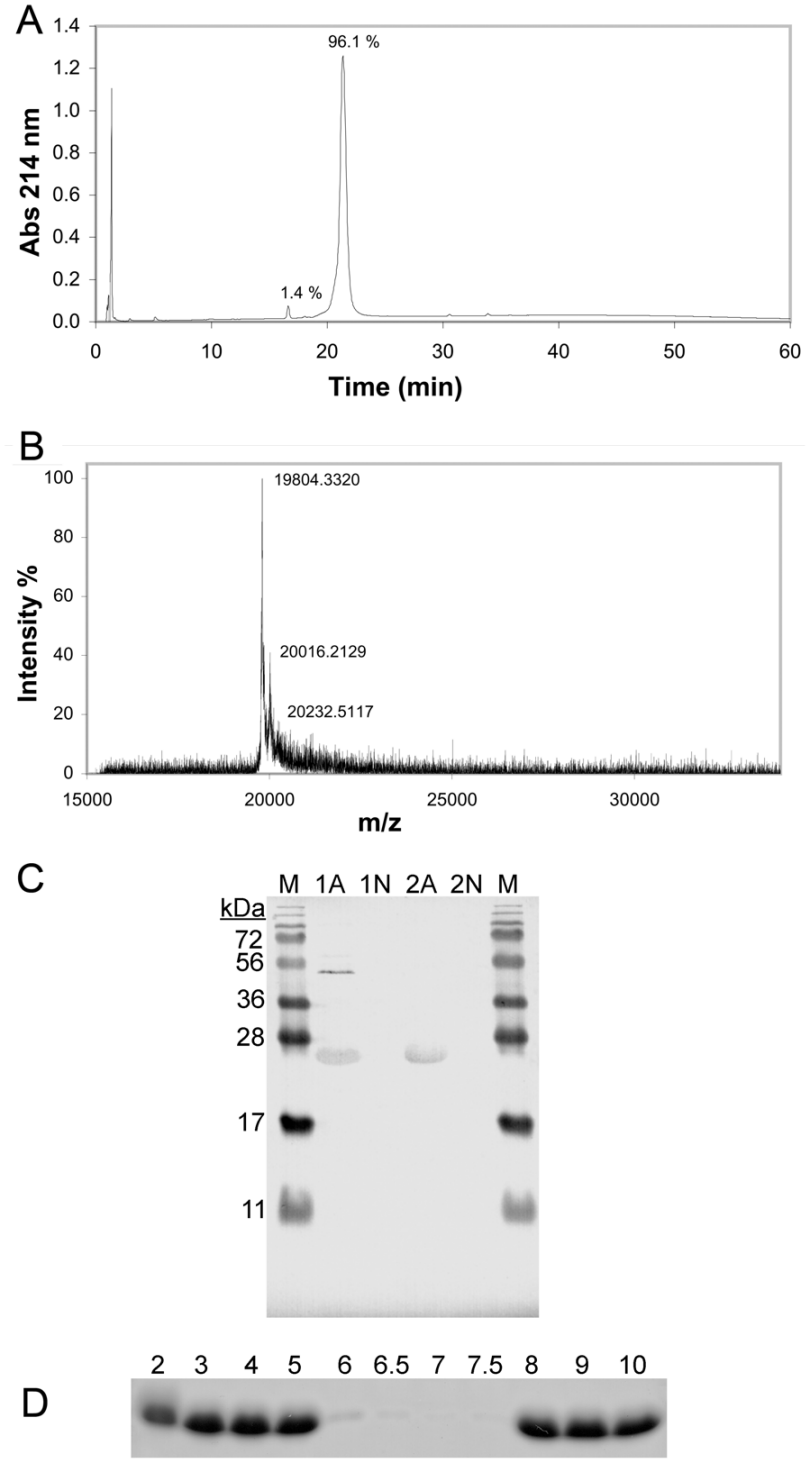
Characterization of recombinant human amelogenin purified using an acid/heat treatment method. **A**) C18 reverse phase HPLC analysis of amelogenin purified with the acid/heat treatment method. Amelogenin elutes at ∼45% acetonitrile and the peak area indicates a purity of >95%. **B**) MALDI-TOF mass spectra of purified amelogenin recorded in linear mode. The obtained mass is very close from expected theoretical mass (19804.8 Da). The peaks seen with slightly higher masses than the main peak is most likely satellite signals derived from matrix adducts that have reacted with the polypeptide. **C**) Western blot of amelogenin samples (A) and negative controls (N). Lanes marked 1 are samples taken from cultivated cells, and lanes marked 2 are from samples after the acid/heat treatment. Lanes marked M contain molecular weight markers. The amelogenin containing samples show a positive reaction not seen in the negative controls, confirming the identity of the protein. The band visible at higher molecular weights in lane 1A suggests that a multimeric form of amelogenin is present inside the cells. **D**) SDS-PAGE analysis of amelogenin solubility test, showing the soluble fractions of purified amelogenin at pH 2–10. The protein is only sparingly soluble at pH 6–7.5, but is readily soluble at more acidic or basic pH.

SDS-PAGE and reversed phase high-performance liquid chromatography (HPLC) analysis both showed a very high purity (>95%) of amelogenin purified only with the acid/heat treatment method (Figure 1 and 2a). A yield of up to ∼1 g/liter shake flask culture could be obtained after a single acid/heat treatment step. MALDI-TOF mass spectrometry analysis (Figure 2b) showed a molecular mass within 2 Da from the expected theoretical mass of the purified amelogenin (19804.8 Da) and Western blot analysis with anti amelogenin antibodies was positive (Figure 2c), confirming the identity of the purified protein.

Amelogenin purified with the acid/heat treatment method was also subjected to a pH solubility test (Figure 2d) and was found to be largely insoluble at pH 6–7.5 and soluble at more acidic or basic pH.

To verify that the purification procedure not had affected the aggregation properties of amelogenin the size of the amelogenin aggregates was measured using dynamic light scattering (DLS). The purified amelogenin formed nanospheres with a hydrodynamic radius (R_H_) of 19.6±4.5 nm at pH 8.0.

### Amelogenin as purification tag

Recombinant amelogenin could be efficiently separated from *E. coli* proteins by a simple heat treatment of the intact cells in 3% HAc. The ease of the process makes it attractive to test also for other proteins/peptides. However, the conditions during purification, with low pH and high temperature, may be too harsh for many proteins. Therefore, a fusion protein approach was tested to see if amelogenin could facilitate purification of other target proteins/peptides using the acid/heat treatment method.

**Figure 3:**
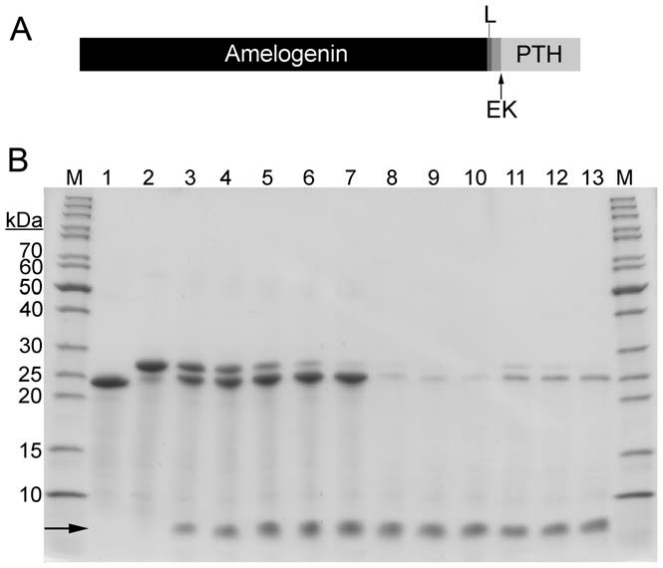
**A**) Schematic structure of the amelogenin-PTH fusion protein. The amelogenin (in the N-terminal) and PTH (in the C-terminal) are separated by a glycine-serine linker (L) and an enterokinase site (EK). The arrow indicates the cleavage point of enterokinase. **B**) SDS-PAGE gel for analysis of amelogenin-PTH cleavage with enterokinase. Lane 1 is amelogenin purified with acid/heat treatment method and lanes 2–7 are purified amelogenin-PTH at different time points during enterokinase cleavage: 0 h, 1 h, 2 h, 4 h, 8 h and 24 h. Lanes 8–10 are samples after 4 h, 8 h and 24 h of cleavage where the amelogenin and amelogenin-PTH have been removed by precipitation at pH 7. Lanes 11–13 are also samples after 4 h, 8 h and 24 h of cleavage, but here the amelogenin and amelogenin-PTH have been removed by precipitation using 250 mM NaCl. Lanes marked M contain molecular weight marker. PTH is indicated with an arrow.

Human parathyroid hormone (PTH), used clinically to treat osteoporosis [33], was used as a model to verify our method. The gene encoding the active part of PTH (PTH1–34) was fused downstream of the amelogenin gene to generate an amelogenin-PTH fusion protein (Figure 3a). Amelogenin and PTH were separated with a glycine-serine linker and an enterokinase cleavage site, allowing for site-specific proteolytic removal of amelogenin. Production of the fusion protein was carried out in the same way as for amelogenin and it was expressed at high levels in *E. coli* BL21 (DE3) cells. The fusion protein could be purified using the acid/heat treatment method, with a similar yield and purity as for amelogenin. In order to disconnect PTH from amelogenin the fusion protein was treated with enterokinase at pH 4.5, which completely cleaved the fusion protein into free amelogenin and PTH. After the cleavage most of the amelogenin could be precipitated from the reaction mixture by raising the pH to ∼7 using NaOH, or by adding NaCl to a concentration of ≥150 mM at pH 4.5. The precipitated amelogenin could then be separated from the soluble PTH by centrifugation (Figure 3b). A plasmid vector containing PTH alone was prepared as a control, but no PTH could be detected in whole cell samples or in the purified samples.

Several other amelogenin fusion proteins have also been successfully produced with the acid/heat treatment method, for instance an amelogenin-ameloblastin fusion protein.

## Discussion

In this report we have described a method for purification of recombinant human amelogenin, by utilizing the special solubility properties of amelogenin. We have also showed that the same method can be used to purify an amelogenin fusion protein (amelogenin-PTH), suggesting that amelogenin can be used as a purification tag for production of other recombinant proteins or peptides.

The described method comprises heat treatment at 80°C of intact amelogenin producing *E. coli* cells suspended in 3% HAc, followed by isolation of the soluble fraction. The number of steps is kept to a minimum and everything from harvesting of the cells to isolation of the purified amelogenin can be carried out in the same vessel. The method should be well suited also for large-scale applications and involves minimal use of chemicals or expensive materials. The thermal lysis used could have several advantages for process scale use, such as product sterilization, protease inactivation and generation of relatively large cell debris which may be more easily separated from the soluble fraction [34]. Some amelogenin was released from the cells even without heat treatment, indicating that some cell disruption occur only as a result of the acidic environment. The method should be seen as a combined cell disruption and product capture/purification step and additional purification steps, e.g. chromatographic techniques, may be necessary depending on the application of the protein. Nevertheless, the described method should be an excellent first step regardless of the application of the final product and is compatible with subsequent purification techniques.

The effect of the purification procedure on the biologic activity of recombinant amelogenin can not be easily assayed since amelogenin lacks suitable measurable activity. However, when our amelogenin preparation was proteolytically digested, it exhibited similar growth stimulating effects on cell cultures as murine amelogenin. Previously, a preparation containing porcine amelogenin (EMD), extensively used in the literature, was also reported withstanding high temperatures without precipitating [35] or losing the bioactivities [36]. The solubility profile and the aggregation properties of the human amelogenin tested in this study is also in line with what has been previously reported for recombinant murine amelogenin [30]–[31], [37]. This shows that amelogenin purified in this work share the same physiochemical characteristics as amelogenin in previous reports purified using the original method by Simmer et al. [30], and is not negatively affected by purification procedures.

The measured mass of the purified amelogenin suggests that the N-terminal methionine is missing in the purified protein, a phenomenon previously reported and suggested to be caused by *E. coli* methionine aminopeptidase [30]. The apparent molecular mass derived from the SDS-PAGE gel is higher than the mass measured with MALDI-TOF mass spectrometry. This slow migration of amelogenin on SDS-PAGE gels have been previously reported and is possibly due to the unusual primary sequence of amelogenin [30].

The results in this report demonstrate a simple method for separating recombinant amelogenin from *E. coli* proteins. This suggests that amelogenin could be used as a purification tag to facilitate purification of other proteins or peptides using the same method. Indeed, an amelogenin-PTH fusion protein was successfully separated from host cell proteins, and isolation of the target protein (PTH) could be carried out by digesting the fusion protein with a site specific protease followed by precipitation of the amelogenin part by adjusting the pH to ∼7 or by increasing the salt concentration.

The solubility of the amelogenin-PTH fusion protein showed a similar profile to that of amelogenin, with a low solubility at physiologic pH (data not shown). This may restrict the choice of method for removal of the amelogenin purification tag, since the fusion protein needs to be soluble to do this. In this work enterokinase was used for specific proteolytic tag removal, since it is active at acidic pH, which circumvents the potential solubility problem.

The results presented here describe a novel method for amelogenin purification, and identify amelogenin as an attractive fusion partner as a carrier protein for other target proteins/peptides. In one step intact host cells are treated to produce a >95% pure amelogenin, with minimal use of chemicals, and the same procedure can also be used to purify amelogenin fusion proteins to a similar purity. This implies that amelogenin not only has use as a therapeutic agent, but also can be used to facilitate production of other important peptides and proteins.

## Materials and Methods

### Gene synthesis

A gene encoding the X-chromosomal human 175 amino acid amelogenin (Swissprot Q99217, isoform 1, excluding the signal peptide) was synthesized by polymerase chain reaction (PCR). Nine oligonucleotides ∼80 bp long with ∼20 bp complementary ends were used to build the gene, which was codon optimized for *E. coli*. The oligonucleotides (0.05 µM of each oligonucleotide in the reaction mixture) were mixed and assembled using PfuUltra Hotstart DNA polymerase (Stratagene). The assembled amelogenin gene was amplified, by using flanking primers and the assembly mixture as template, and subsequently cloned into a cloning vector. The gene was sequenced and point mutations were corrected with QuickChange Site-Directed Mutagenesis Kit (Stratagene). The gene was finally inserted between the *Nde*I and *Bam*HI sites in the expression vector pET11a (Novagen). For the gene encoding human PTH1-34 two ∼80 bp oligonucleotides were used and the gene assembly, amplification and cloning procedures were the same as for amelogenin.

### Construction of amelogenin-PTH fusion protein

The genes encoding amelogenin and PTH were individually amplified by PCR. The primers were designed to add extra complementary sequence downstream of the amelogenin gene and upstream of the PTH gene. The complementary sequence encoded a glycine-serine linker and an enterokinase site (Asp-Asp-Asp-Asp-Lys). The two genes could be assembled, utilizing the complementary sequence, and subsequently amplified with flanking primers using PfuUltra Hotstart DNA polymerase. The amelogenin-PTH gene was subsequently cloned into a pET11 expression vector the *Nde*I and *Bam*HI sites.

### Cultivation

All cultivation was carried out in shake flasks using the *E. coli* expression strain BL21 (DE3), transformed with the proper expression vector. The cultivations were carried out at 37°C in a shaking incubator using TB-medium (tryptone 12 g/l, yeast extract: 24 g/l, glycerol: 4 ml/l, 17 mM KH_2_PO_4_, 72 mM K_2_HPO_4_) containing 200 µg/ml ampicillin. To inoculate 100 ml TB-medium 0.5–1 ml overnight culture was used and the cultivations were induced with 5 mM lactose 4 hours post inoculum. Sampling for SDS-PAGE was carried out by measuring OD_600_, pellet 250 µl cell culture by centrifugation, and resuspending (and boil) the cells in SDS-PAGE loading buffer to a cell density equivalent to an OD_600_ of 10.

### Acid/heat treatment purification

Cells cultivated for a total of 24 hours were harvested by centrifugation and washed in 150 mM NaCl to remove growth medium components. The cell pellets were then resuspended in 3% acetic acid (30 ml HAc to cells from 100 ml cell culture). The cell suspension was then subjected to sonication (8×10 seconds), or to 20 minutes heat treatment at 60 or 80°C in a water bath, followed by centrifugation at 20 000 g for 20 minutes. The supernatant from the sonicated sample was also heat treated and re-centrifuged as above. All supernatants were collected and analyzed. The samples were kept at 4–8°C during all steps except for the heat treatment. The heat treatment of the cells at 80°C was repeated, in new experiments, using different concentrations of HAc (0.5–5%). Different volumes of 3% HAc were also tested (6–60 ml to cells from 100 ml cultivation). The protein concentration in the supernatants was measured using Bio-Rad Protein Assay (Bio-Rad).

### SDS-PAGE and Western blot

SDS-PAGE analysis was carried out on 10–20% gradient gels stained with Coomassie brilliant blue. For Western blot a 15% gel was electroblotted to a nitrocellulose membrane which was subsequently blocked with 3% nonfat milk powder. The membrane was incubated with a primary antibody against recombinant human amelogenin (kindly supplied Straumann Biologics, Malmö, Sweden) and then with a secondary horse radish peroxidase conjugated antibody. The membrane was developed with HRP Color Development Reagent (Bio-Rad).

### Solubility test

Amelogenin purified with the acid/heat treatment method was dialyzed against 0.05% acetic acid adjusted to pH 4 with NaOH, and concentrated ∼10 times using Vivaspin 6 ultrafiltration membrane (Sartorius). Five µl of the concentrated sample was mixed with 95 µl of 0.1 M buffers with pH ranging from 2 to 10. The samples were then incubated 1 hour at room temperature and centrifuged 15 minutes as 16 000 g. The supernatant containing the soluble fraction was analyzed by SDS-PAGE.

### Dynamic light scattering

Amelogenin samples (5 mg/ml in 100 mM glycine, pH 8.0), were analyzed at 20°C using a DynaPro instrument from ProteinSolutions equipped with a microsampler temperature control unit. The data obtained, were analyzed using Dynamics software (ver 5.24.02).

### HPLC

Soluble fraction generated from the acid/heat treatment was analyzed on a 12.5 cm C18 reversed phase HPLC column (5 µm particle size). The analysis was carried out with a 60 minutes linear gradient starting with 90% solvent A (0.1% TFA in H_2_O) and 10% solvent B (0.1% TFA in acetonitrile) and ending with 100% solvent B. Detection was carried out by measuring the absorbance at 214 nm.

### Mass spectroscopy

Amelogenin purified with the acid/heat treatment method was diluted with 70% acetonitrile containing 0.1% TFA to a concentration of ∼10 pmol/µl. The MALDI sample was prepared by adding 0.5 µl of 10 mg/ml sinapinic acid (dissolved in 50% acetonitrile, 0.05% TFA) to the MALDI sample plate, and when the matrix solution had dried 0.5 µl of the amelogenin solution was added to the matrix spots. The dried sample was analyzed in linear mode with an externally calibrated Applied Biosystems 4700 Proteomics Analyzer.

### Enterokinase cleavage of amelogenin-PTH fusion

The fusion protein was purified in 3% HAc according to the acid/heat treatment method described above. The cleavage was carried out in 100 mM HAc pH 4.5, 1 mM CaCl_2_ at room temperature using ∼1 U/mg fusion of EKMax Enterokinase (Invitrogen). The reaction was incubated for 24 hours and samples were taken at different time points for analysis with SDS-PAGE. Samples were also taken out where the amelogenin and amelogenin-PTH were precipitated by adding 2.5 M NaOH until pH was ∼7, or by adding NaCl to a concentration of ≥150 mM. The precipitate was removed by centrifugation and the supernatant containing PTH was analyzed by SDS-PAGE.
